# Normal tissue adjacent to tumor expression profile analysis developed and validated a prognostic model based on Hippo‐related genes in hepatocellular carcinoma

**DOI:** 10.1002/cam4.3890

**Published:** 2021-04-04

**Authors:** Qingbo Pan, Fanbo Qin, Hanyu Yuan, Baoning He, Ni Yang, Yitong Zhang, Hong Ren, Yi Zeng

**Affiliations:** ^1^ Department of Infectious Diseases The Key Laboratory of Molecular Biology for Infectious Diseases Chinese Ministry of Education Institute for Viral Hepatitis The Second Affiliated Hospital of Chongqing Medical University Chongqing China; ^2^ Department of Hepatobiliary Surgery The Second Affiliated Hospital of Chongqing Medical University Chongqing China; ^3^ Caojie Community Medical Service Centre Hechuan Chongqing China; ^4^ Chongqing YuCai Secondary School Chongqing China

**Keywords:** hepatocellular carcinoma, Hippo‐related genes, paired normal tissues adjacent to HCC, prognosis

## Abstract

**Background:**

Hepatocellular carcinoma (HCC) is the most common malignant disease worldwide. Although the diagnosis and treatment of HCC have greatly improved in the recent years, there is still a lack of accurate methods to predict the prognosis of patients. Evidence has shown that Hippo signaling in tissues adjacent to HCC plays a significant role in HCC development. In the present study, we aimed to construct a model based on the expression of Hippo‐related genes (HRGs) in tissues adjacent to HCC to predict the prognosis of HCC patients.

**Methods:**

Gene expression data of paired normal tissues adjacent to HCC (PNTAH) and clinical information were obtained from Gene Expression Omnibus (GEO) and The Cancer Genome Atlas (TCGA) databases. The HRG signature was constructed using four canonical Hippo‐related pathways. Univariate Cox regression analysis was used to screen survival‐related HRGs. LASSO and multivariate Cox regression analyses were used to construct the prognostic model. The true and false positive rates of the model were confirmed using receiver operating characteristic (ROC) analysis.

**Results:**

The prognostic model was constructed based on the expression levels of five HRGs (NF2, MYC, BIRC3, CSNK1E, and MINK1) in PNTAH. The mortality rate of HCC patients increased as the risk score determined by the model increased. Furthermore, the risk score was found to be an independent risk factor for the survival of patients. ROC analysis showed that the prognostic model had a better predictive value than the other conventional clinical parameters. Moreover, the reliability of the prognostic model was confirmed in TCGA‐LIHC cohort. A nomogram was generated to predict patient survival. An exploration of the predictive value of the model in HCC tissues indicated that the model is PNTAH‐specific.

**Conclusions:**

We developed and validated a prognostic model based on the expression levels of five HRGs in PNTAH, and this model should be helpful in predicting the prognosis of patients with HCC.

## Graphical abstract

We built a prognostic model based on the Hippo signaling for the first time, and found that the expression of the Hippo signaling in adjacent tissues can better predict the survival of liver cancer patients.

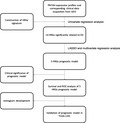

## 1 INTRODUCTION

Hepatocellular carcinoma (HCC), the most frequent primary liver cancer, is ranked sixth among all cancers worldwide.^1^ The 5‐year survival rate for HCC is only 18%, making it the fourth leading cause of cancer‐related deaths.^2^ Although etiological agents responsible for most HCC cases, such as alcoholism and HBV and HCV infection, are well known, the molecular pathogenesis of HCC is not clear.^3^, ^4^, ^5^ Currently, the best treatment for early HCC is surgical excision or liver transplantation.^6^ However, most patients are already in the advanced stages of the tumor when they receive treatment.^7^ Approximately 60% of the patients experience recurrence or distant metastasis after surgery.^8^ Therefore, establishing an effective model to predict the prognosis of patients with HCC can provide new guidance for clinical management.

Currently, the gene expression profiles of not only the tumors of all major types but also of the paired normal tissues adjacent to the tumor, from tens of thousands of patients, are available, such as those in TCGA and GEO. Paired normal tissues adjacent to tumor (PNTAT) are often used as a normal control for cancer research because of the shortage of healthy samples. However, whether PNTAT is truly “normal” is controversial. Recent studies have shown that the transcriptomic profiles of PNTAT are distinct from those of healthy and tumor tissues.^9^ The interaction between PNTAT and tumor may help shape the tumor's microenvironment, indicating an important role of PNTAT in cancer progression.^10^

Hippo signaling was primarily discovered for its control of organ size in Drosophila and is highly conserved in mammals.^11^, ^12^ It has been reported that Hippo signaling is a critical regulator of normal and malignant liver development.^13^ A recent study demonstrated that Hippo signaling was activated in peritumoral hepatocytes to induce tumor regression when primary liver tumors and metastases were present.^14^ Thus, we were interested in determining whether Hippo signaling in paired normal tissues adjacent to HCC (PNTAH) can be a potential molecular prognostic marker.

In this study, we first constructed a Hippo‐related gene (HRG) signature using four canonical Hippo‐related pathways. We then identified 14 prognostic HRGs based on PNTAH expression profiles from GSE14520 using univariate Cox regression analysis. In addition, we screened out five key HRGs (NF2, MYC, BIRC3, CSNK1E, and MINK1) to construct a prognostic model using least absolute shrinkage and selection operator (LASSO) and multivariate Cox regression analyses. We further confirmed that the model is good at predicting prognosis through survival and ROC curve analysis and found that the risk score calculated by the model formula was an independent risk factor through univariate and multivariate Cox regression analyses. Finally, we generated a nomogram to provide clinicians with a quantitative method for predicting survival. Moreover, the reliability of this model was validated by analyzing the PNTAH expression profiles from TCGA‐LIHC. Exploration of the predictive value of these five HRGs and the constructed model in HCC tissues demonstrated that the model is PNTAH‐specific. In summary, the present study may help reveal the underlying role of Hippo signaling in PNTAH and provide a useful prediction tool for HCC survival.

## 2 METHODS

### 2.1 Data acquisition

Expression profiles from the GSE14520 dataset (232 HCC samples, 232 PNTAH samples, and corresponding clinical data), GSE102079 dataset (14 normal liver samples, NL samples), and GSE112790 dataset (15 NL samples) were downloaded from the GEO database (www.pubmed.com/geo). The ComBat function from the sva package in R was used to remove the batch effects of the three datasets. TCGA‐GTEx cohort was downloaded from the UCSC Xena browser (http://xena.ucsc.edu/), among which 110 normal liver samples of GTEx, 50 PNTAH samples, 371 HCC samples, and the corresponding clinical data of TCGA liver cancer hepatocellular carcinoma (TCGA‐LIHC) were selected for the next analysis. To make data from different sources more compatible, the UCSC Xena project recomputed all expression raw data based on a standard pipeline to minimize differences from distinct sources. Principal component analysis (PCA) was applied using the PCA function implemented in the FactoMineR package. The 232 PNTAH samples from GSE14520 were used to construct the prognostic model, and 50 PNTAH samples from TCGA‐LIHC were used for validation.

### 2.2 Construction of HRG signature and function enrichment

Four Hippo‐related canonical pathways, GO_HIPPO_SIGNALING, KEGG_HIPPO_SIGNALING_PATHWAY, REACTOME_SIGNALING_BY_HIPPO and WP_HIPPOYAP_SIGNALING_PATHWAY, were obtained from the Molecular Signature Database (MsigDB, http://software.broadinstitute.org/gsea/msigdb/). After merging the four canonical pathways, a Hippo‐related signature was constructed. The heatmap was plotted using the pheatmap package in R to show the expression levels of the HRG signature in different samples. The protein–protein interaction (PPI) of the HRGs was predicted using STRING (https://string‐db.org/) and visualized using Cytoscape (v3.7.2). Gene Ontology (GO) and Kyoto Encyclopedia of Genes and Genomes (KEGG) pathway analyses were carried out using the R clusterProfiler package. Enrichment results were visualized using the enrichment plot package. A *p*‐value <0.05 was set as the cutoff criterion for both GO and KEGG functional analysis.

### 2.3 Construction and validation of prognostic model using PNTAH expression profiles

To screen out the prognostic HRGs, the association between PNTAH HRG expression and overall survival (OS) was evaluated using univariate Cox regression. Genes with a *p*‐value < 0.05 were considered prognosis‐related HRGs. The differential expression of the prognostic HRGs among NL, PNTAH, and HCC was determined using the Kruskal–Wallis test. Then, LASSO and multivariate Cox regression analyses were conducted to identify key prognostic HRGs. Finally, a prognostic model was constructed, and the risk score formula was as follows: risk score = (the expression of gene_1_ in PNTAH ×regression coefficient of gene_1_) + (the expression of gene_2_ in PNTAH × regression coefficient of gene_2_) + … + (the expression of gene_n_ in PNTAH × regression coefficient of gene_n_).

Based on the median risk score calculated using the PNTAH expression profiles, the patients were divided into low‐ or high‐risk groups. The difference in OS between the two groups was analyzed using the Kaplan–Meier method and log‐rank test. The risk score distribution, number of patients examined, and the heatmap of the prognostic HRGs in different risk groups were displayed. Univariate and multivariate Cox regression analyses were performed to explore whether the risk score could be an independent indicator of OS. The true and false positive rates of the prognostic model were analyzed using the receiver operating characteristic (ROC) and the area under the curve (AUC). A nomogram was constructed to estimate the 1‐, 3‐, and 5‐year survival rates of HCC patients using the rms package in R, and calibration of the nomogram was measured using calibration curves. Moreover, the predictive value of the constructed model was further confirmed using independent data from TCGA‐LIHC.

### 2.4 Verification of key HRGs and the prognostic model using HCC tissue expression profiles

To investigate whether the prognostic model built using PNTAH also had prognostic value in HCC tissues, the expression profiles of HCC tissues were extracted from GSE14520. HCC patients were divided into high‐ or low‐expression groups based on the median expression of the key HRGs in HCC tissues. Moreover, the risk score was calculated with the established formula for PNTAH, using the HCC tissue expression profiles, and the patients were divided into high‐ or low‐risk groups based on the median risk score. The survival curve was drawn using the Kaplan–Meier method, and the difference in the survival rate between different groups was verified by the log‐rank test. Similarly, the prognostic value of the key HRGs and the constructed model for the HCC tissues of TCGA‐LIHC cohort were also analyzed.

### 2.5 Statistical analysis

Statistical analysis was carried out using R 4.0.1 (https://www.r‐project.org). Univariate Cox regression analysis was conducted to estimate prognosis‐related HRGs. The Kruskal–Wallis rank sum test was used to determine whether the HRGs were differentially expressed among NL, PNTAH, and HCC. LASSO regression analysis was used to prevent overfitting. Multivariate Cox regression analysis was performed to construct a prognostic model. An independent *t*‐test was performed to analyze the association between the risk score and conventional clinical characteristics. A nomogram was created using the rms package in R. ROC analysis was performed to test the true and false positive rates of the model. The survival curve was plotted using the survival and survminer package of R. Forest maps were plotted using the forsetplot package of R. The survivalROC package was used to generate the ROC curves, and the AUC values were calculated according to the ROC curves. All tests were two‐tailed and considered significant when *p* < 0.05.

## 3 RESULTS

### 3.1 **Differential gene expression pattern of NL**, **PNTAH**, **and HCC**

**FIGURE 1:**
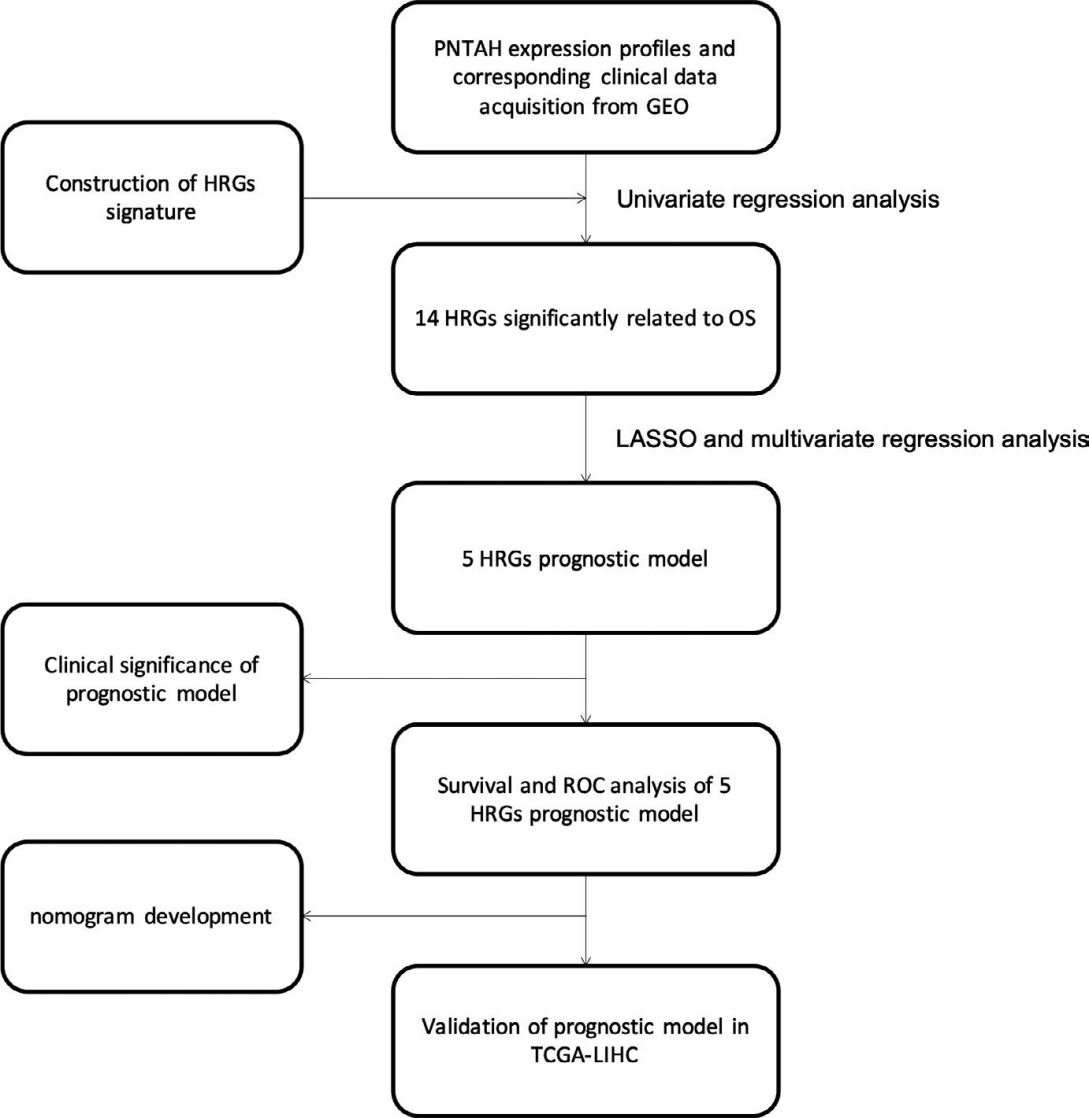
Workflow of this study. PNTAH, paired normal tissues adjacent to HCC; GEO: Gene Expression Omnibus Database; OS: overall survival; LASSO: Least Absolute Shrinkage and Selection Operator; HRGs, Hippo‐related genes; ROC, receiver operating characteristic curve; TCGA‐LIHC: TCGA Liver Cancer Hepatocellular Carcinoma.

**FIGURE 2:**
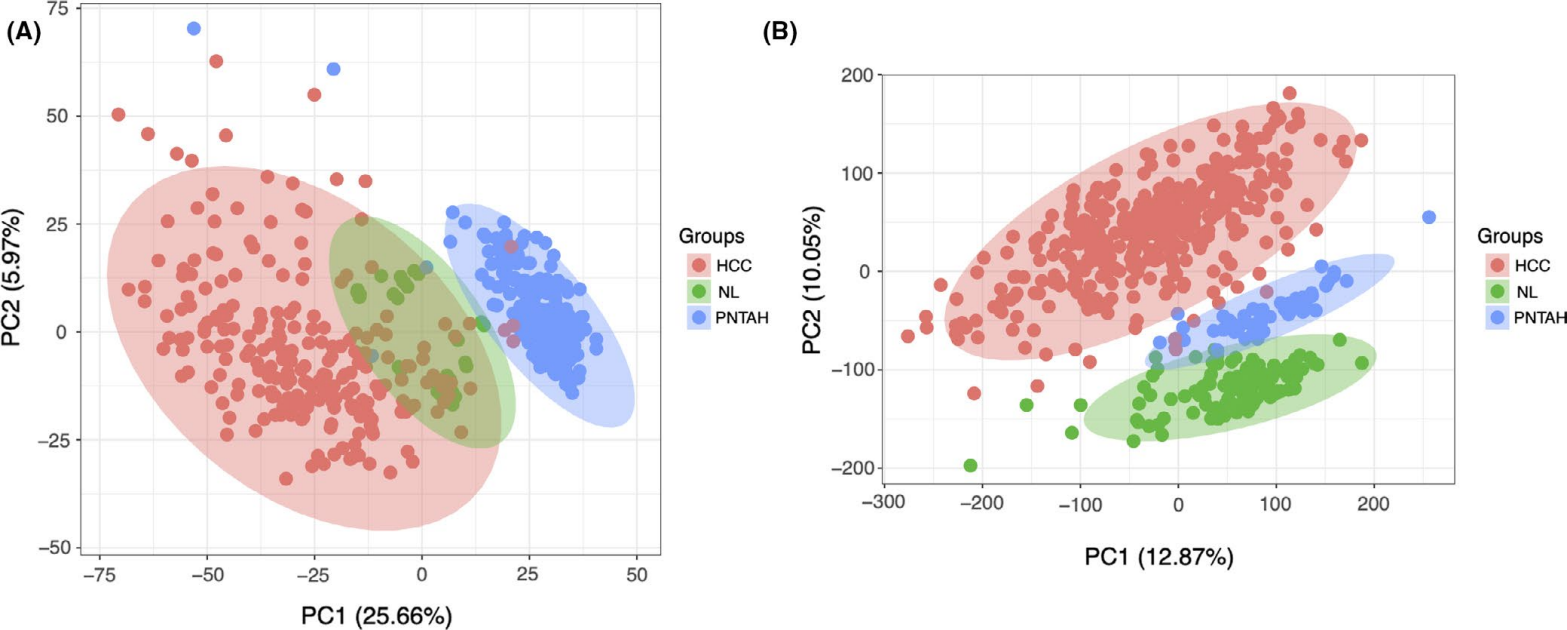
Differential gene expression pattern of NL, PNTAH, and HCC. (A) Principal‐component analysis (PCA) of gene expression pattern of NL, PNTAH, and HCC in GEO cohort. (B) PCA of gene expression pattern of NL, PNTAH, and HCC in TCGA‐GTEx cohort. NL, normal liver; PNTAH, paired normal tissues adjacent to HCC; HCC, hepatocellular carcinoma. PCA1 and PCA2 represent the top two dimensions of gene expression in each group.

A total of 29 NL samples, 232 PNTAH samples, and 232 HCC samples with mRNA expression profiles were obtained from the GEO database. The available clinical characteristics of 232 HCC patients, including age, gender, tumor stage, tumor size, recurrence status, survival time, and survival status, are presented in Table 1. A diagram presenting the workflow of the prognostic model starting from data acquisition to statistical analysis strategies is given in Figure 1. After removing the batch effect, PCA was performed to analyze the expression patterns of the different samples. The first principal component (PCA1) and the second principal component (PCA2) explained 25.66% and 5.97% of the variation in the data, respectively. The mRNA expression profile was scattered into three different clusters, which revealed striking differences among the genes in NL, PNTAH, and HCC in the GEO cohort (Figure 2A), and this pattern was also observed in TCGA‐GTEx cohort (110 NL samples, 50 PNTAH samples, and 371 HCC samples) (Figure 2B).

**TABLE 1:** Summary of clinical data.

	GEO (*N* = 232)	TCGA (*N* = 50)	Overall (*N* = 282)
Gender			
Male	200 (86.2%)	28 (56.0%)	228 (80.8%)
Female	27 (11.6%)	22 (44.0%)	49 (17.4%)
Unknown	5 (2.2%)	0 (0%)	5 (1.8%)
Age (years)			
Median [Min, Max]	51.0 [21.0, 77.0]	61.7 [20.0, 81.0]	52.9 [20.0, 81.0]
Tumor stage			
Ⅰ	93 (40.0%)	18 (36.0%)	111 (39.4%)
Ⅱ	74 (31.9%)	11 (22.0%)	85 (30.1%)
Ⅲ	44 (19.0%)	12 (24.0%)	56 (19.9%)
Ⅳ	0 (0%)	1 (2.0%)	1 (0.3%)
Unknown	21 (9.1%)	8 (16.0%)	29 (10.3%)
Tumor size			
<=5 cm	82 (35.3%)	NA	82 (35.3%)
>5 cm	144 (62.1%)	NA	144 (62.1%)
Unknown	6 (2.6%)	NA	6 (2.6%)
Recurrence status			
Recurrence	126 (54.3%)	NA	126 (54.3%)
noRecurrence	101 (43.5%)	NA	101 (43.5%)
Unknown	5 (2.2%)	NA	5 (2.2%)
Survival status			
Death	88 (37.9%)	34 (68.0%)	122 (43.3%)
Alive	139 (59.9%)	16 (32.0%)	155 (55.0%)
Unknown	5 (2.2%)	0 (0%)	5 (1.7%)
Survival time (days)			
Median [Min, Max]	1242.4[55.0, 2089]	962.2 [11.0, 3437]	1039.2 [11.0, 3437]

Abbreviation: NA, not available data.

### 3.2 Construction of HRG signature and functional enrichment

**FIGURE 3:**
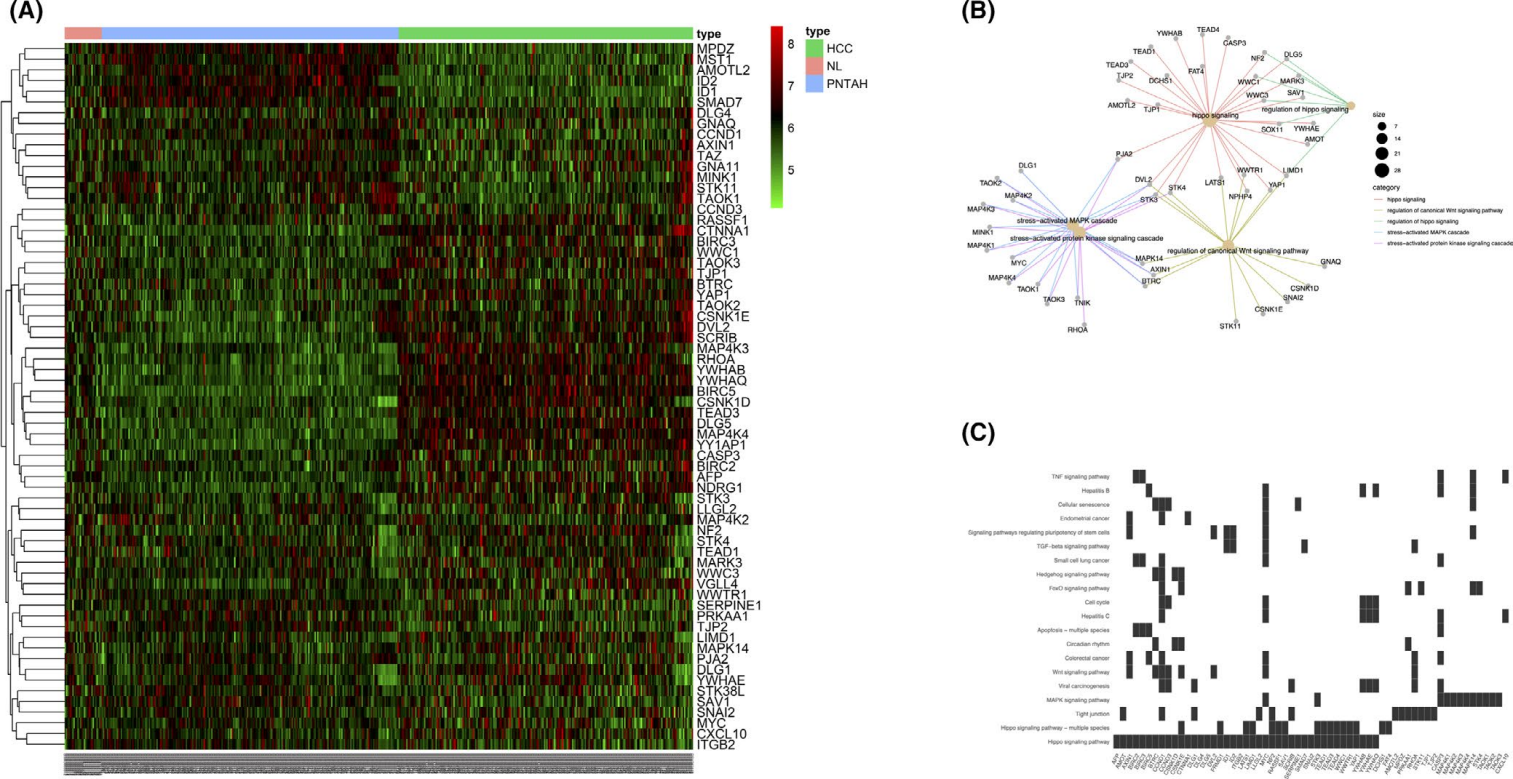
Construction of HRG signature. (A) The heatmap of the HRG expression levels in different groups. The color of each block depends on the expression value. (B) Gene Ontology (GO) enrichment analysis of the HRG signature. (C) Kyoto Encyclopedia of Genes and Genomes (KEGG) analysis of the HRG signature. HRG: Hippo‐related genes.

To construct an HRG signature, four Hippo‐related canonical pathways GO_HIPPO_SIGNALING, KEGG_HIPPO_SIGNALING_PATHWAY, REACTOME_SIGNALING_BY_HIPPO and WP_HIPPOYAP_SIGNALING_PATHWAY, were downloaded from MsigDB (Table 2). The above gene sets were mainly curated from pathway databases, biomedical literature, and individual domain experts. Finally, a total of 76 genes were identified as HRGs after removing duplicates and missing probes in the expression profiles. The PPI network of these 76 HRGs visualized using Cytoscape is shown in Figure S1. The heatmap of these 76 HRGs indicated that Hippo signaling may be differentially regulated among NL, PNTAH, and HCC (Figure 3A). The GO functional enrichment analysis of the 76 HRGs is presented in Table 3. These HRGs were related to Hippo signaling, regulation of Hippo signaling, regulation of canonical Wnt signaling pathway, stress‐activated MAPK cascade, and stress‐activated protein kinase signaling cascade (Figure 3B). The KEGG pathways were mainly enriched in the Hippo signaling pathway and tight junction and MAPK signaling pathways (Figure 3C).

**TABLE 2:** Construction of Hippo‐related gene set.

Gene set	
GO_HIPPO_SIGNALING	AJUBA,AMOT,AMOTL1,AMOTL2,CASP3,DCHS1,DLG5,DVL2,FAT4,FRMD1,IQCJ‐SCHIP1,LATS1,LATS2,LIMD1,MAP2 K3,MAPK14,MARK3,MOB1A,MOB1B,MOB3B,NEK8,NF2,NPHP4,PJA2,SAV1,SCHIP1,SOX11,STK3,STK4,TEAD1,TEAD2,TEAD3,TEAD4,TJP1,TJP2,VGLL4,WTIP,WWC1,WWC2,WWC3,WWTR1,YAP1,YWHAB,YWHAE
KEGG_HIPPO_SIGNALING_PATHWAY	CTGF,AREG,GLI2,BIRC5,AFP,ITGB2,GF1,SMAD7,SERPINE1,ID1,ID2,AXIN1,AXIN2,NKD1,MYC,SOX2,SNAI2,BIRC2,BIRC3,CCND1,CCND2,CCND3,BBC3,YAP1,TAZ
REACTOME_SIGNALING_BY_HIPPO	AMOT,AMOTL1,AMOTL2,CASP3,DVL2,LATS1,LATS2,MOB1A,MOB1BNPHP4,SAV1,STK3,STK4,TJP1,TJP2,WWC1,WWTR1,YAP1,YWHAB,YWHAE
WP_HIPPOYAP_SIGNALING_PATHWAY	CXCL10,LATS1,LATS2,MAP4 K1,MAP4 K2,MAP4 K3,MAP4 K4,MINK1,MST1,NDRG1,NF2,RASSF1,SAV1,STK3,STK38L,TAZ,TEAD1,TEAD2,TEAD3,TEAD4,TNIK,YWHAQ,YY1AP1

**TABLE 3:** Top 10 enrichment results of HRGs.

Category	Description	Count	*p*‐value
GO	Hippo signaling	8	3.98E−19
	Regulation of canonical Wnt signaling pathway	4	4.78E−05
	Canonical Wnt signaling pathway	4	8.84E−05
	Regulation of Wnt signaling pathway	4	1.21E−04
	Negative regulation of protein phosphorylation	4	2.29E−04
	Negative regulation of phosphorylation	4	3.20E−04
	Transcription regulator complex	4	1.64E−04
	Regulation of hippo signaling	3	7.36E−08
	Regulation of metanephros development	3	5.89E−07
	Regulation of kidney development	3	9.09E−06
KEGG	Hippo signaling pathway	41	6.73E−56
	Hippo signaling pathway—multiple species	16	2.62E−27
	Tight junction	13	5.89E−10
	MAPK signaling pathway	12	3.07E−06
	Viral carcinogenesis	9	3.28E−05
	Wnt signaling pathway	8	3.84E−05
	Human papillomavirus infection	8	4.79E−03
	PI3 K‐Akt signaling pathway	8	7.13E−03
	Hepatitis C	7	2.47E−04
	Human cytomegalovirus infection	7	2.08E−03

Abbreviations: GO, Gene Ontology; HRG, Hippo‐related gene; KEGG, Kyoto Encyclopedia of Genes and Genomes.

### 3.3 Construction of the HRG‐based prognostic model using PNTAH expression profiles of GEO cohort

**FIGURE 4:**
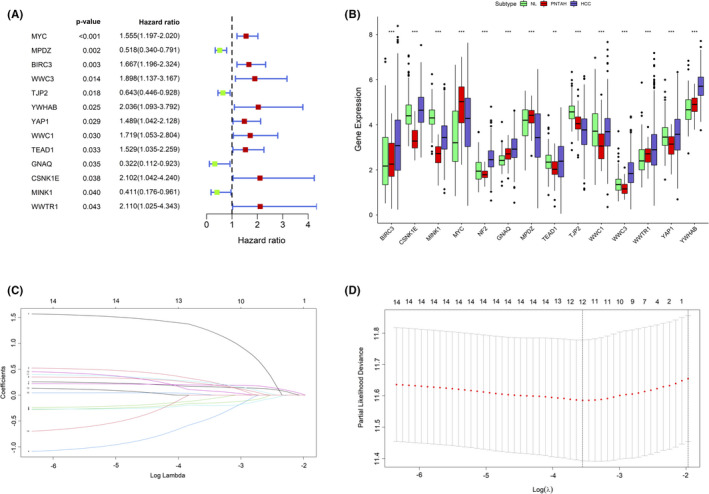
Univariate Cox regression and LASSO regression analysis. (A) Fourteen HRGs with prognostic value determined by univariate Cox regression. NF2 was excluded due to the wide range of the hazard ratio. (B) The boxplot for the expression of 14 prognosis‐related HRGs in NL, PNTAH, and HCC. (C) The changing trajectory of each independent variable. (D) Confidence intervals for each lambda. HRGs: Hippo‐related genes; NL, normal liver; PNTAH, paired normal tissues adjacent to HCC; HCC, hepatocellular carcinoma. LASSO: Least Absolute Shrinkage and Selection Operator. *P value <0.05; **P value <0.01; ***P value <0.001; ns, no significance.

To identify prognostic HRGs expressed in PNTAH, univariate Cox regression analysis was applied with the criteria of *p*‐value < 0.05. Subsequently, 14 genes in PNTAH were selected and found to be significantly related to the OS of patients (Figure 4A). Meanwhile, hazard ratio (HR) and 95% confidence intervals (CIs) were estimated and displayed. Among them, NF2 significantly correlated with survival and had the highest HR (HR = 10.993, 95% CI = 1.367–88.438, *p* = 0.024), suggesting that the expression of NF2 in PNTAH was of great significance in HCC patients. However, since the HR of NF2 was much higher than that of other genes, it was temporarily excluded from the forest map of the univariate regression analysis. In addition, these 14 genes were found to be differentially expressed among NL, PNTAH, and HCC (Figure 4B).

Based on the prognosis‐related HRGs in PNTAH, LASSO and multivariate Cox regression analyses were conducted to construct the HRG‐based model (Figure 4C,D). Five genes, NF2, MYC, CSNK1E, BIRC3, and MINK1, were finally selected to construct the model (Table 4), and the risk score formula for the model was risk score = (0.369537168 × BIRC3 expression in PNTAH) + (0.727258146 × CSNK1E expression in PNTAH) + (−0.885485399 × MINK1 expression in PNTAH) + (0.308063532 × MYC expression in PNTAH) + (2.506094696 × NF2 expression in PNTAH).

**TABLE 4:** Multivariate Cox regression analysis.

Gene	Coefficients	HR	HR.95L	HR.95H	*p*‐value
BIRC3	0.369537168	1.447064711	1.003797478	2.086074458	0.047672596
CSNK1E	0.727258146	2.069398832	0.892533363	4.798040843	0.090077605
MINK1	−0.885485399	0.412513891	0.158456116	1.073910646	0.069694068
MYC	0.308063532	1.36078744	1.029154239	1.799285653	0.030648489
NF2	2.506094696	12.25696928	1.299938769	115.5695172	0.028589509

Abbreviations: HR, hazard ratio; HR.95H, hazard ratio 95% CI high; HR.95L, hazard ratio 95% CI low.

### 3.4 Validation of the constructed model in PNTAH

**FIGURE 5:**
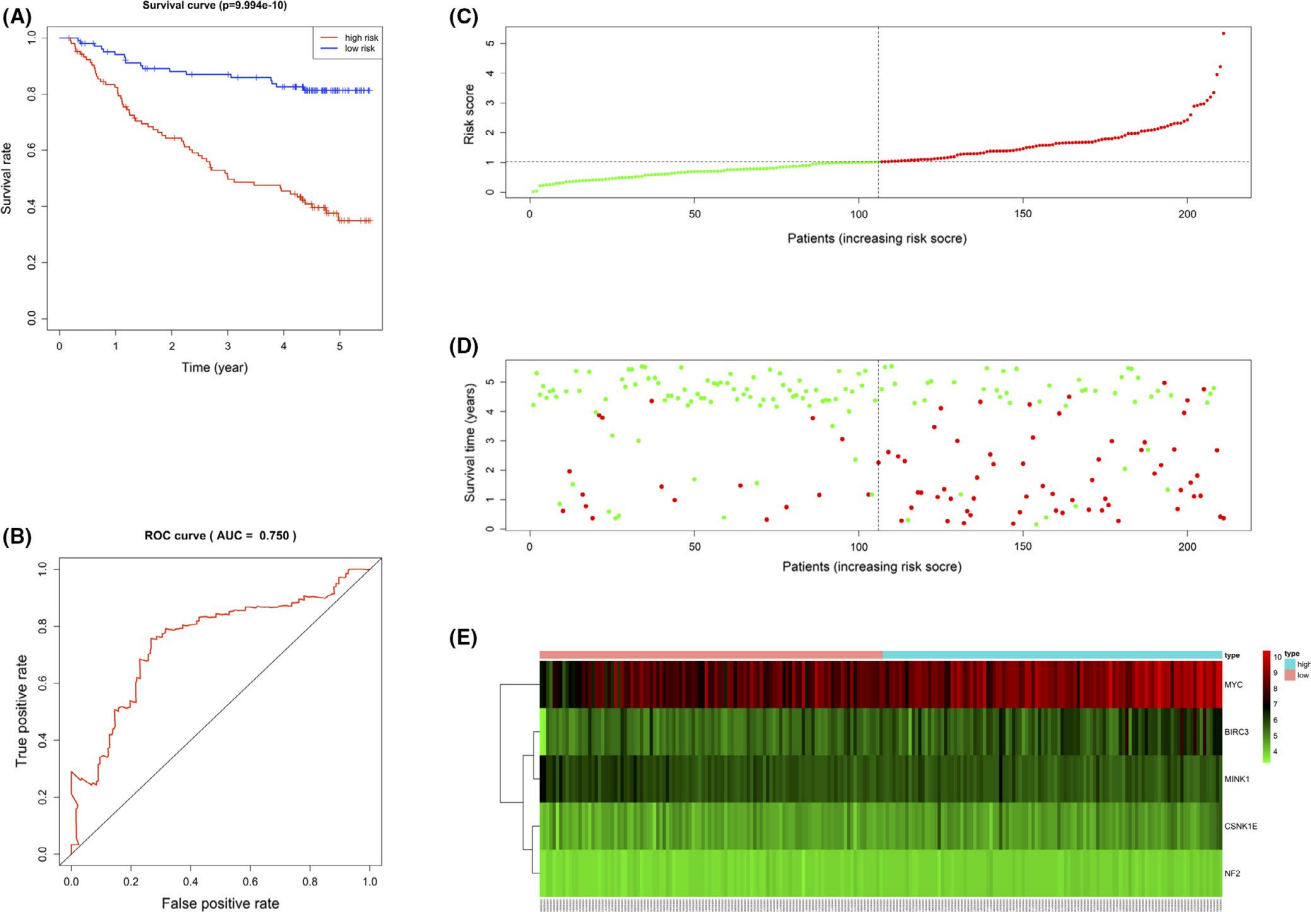
Evaluation of the HRG‐based prognostic model using PNTAH expression profiles from GSE14520. (A) Kaplan–Meier curve analysis of the high‐risk and low‐risk groups. (B) Time‐dependent ROC curve analysis of the prognostic model. (C) The risk score distribution of patients in the prognostic model. (D) Survival status scatter plots for patients in the prognostic model. (E) Expression patterns of risk genes in the prognostic model. PNTAH, paired normal tissues adjacent to HCC; ROC, receiver operating characteristic curve.

To assess the performance of the prognostic model in predicting the clinical outcomes of patients, the risk score of each HCC patient was calculated based on the PNTAH expression profiles and the patients were divided into high‐ or low‐risk groups according to the median risk score. Twenty‐two patients with HCC with missing clinical data were excluded. The median risk score was 1.0278. The survival curve indicated that the high‐risk group (*n* = 105) had a lower survival rate than the low‐risk group (*n* = 105) (Figure 5A). The AUC of the ROC curve was 0.750, proving that the prognostic model performed well for survival prediction (Figure 5B). The risk score distribution, survival status of each patient, and the heatmap of five gene expression profiles in the GSE14520 PNTAH samples are shown (Figure 5C–E). The results illustrated that the survival time decreased and the mortality rate increased as the risk score increased.

### 3.5 Further validation of the prognostic model using PNTAH expression profiles of TCGA‐LIHC

**FIGURE 6:**
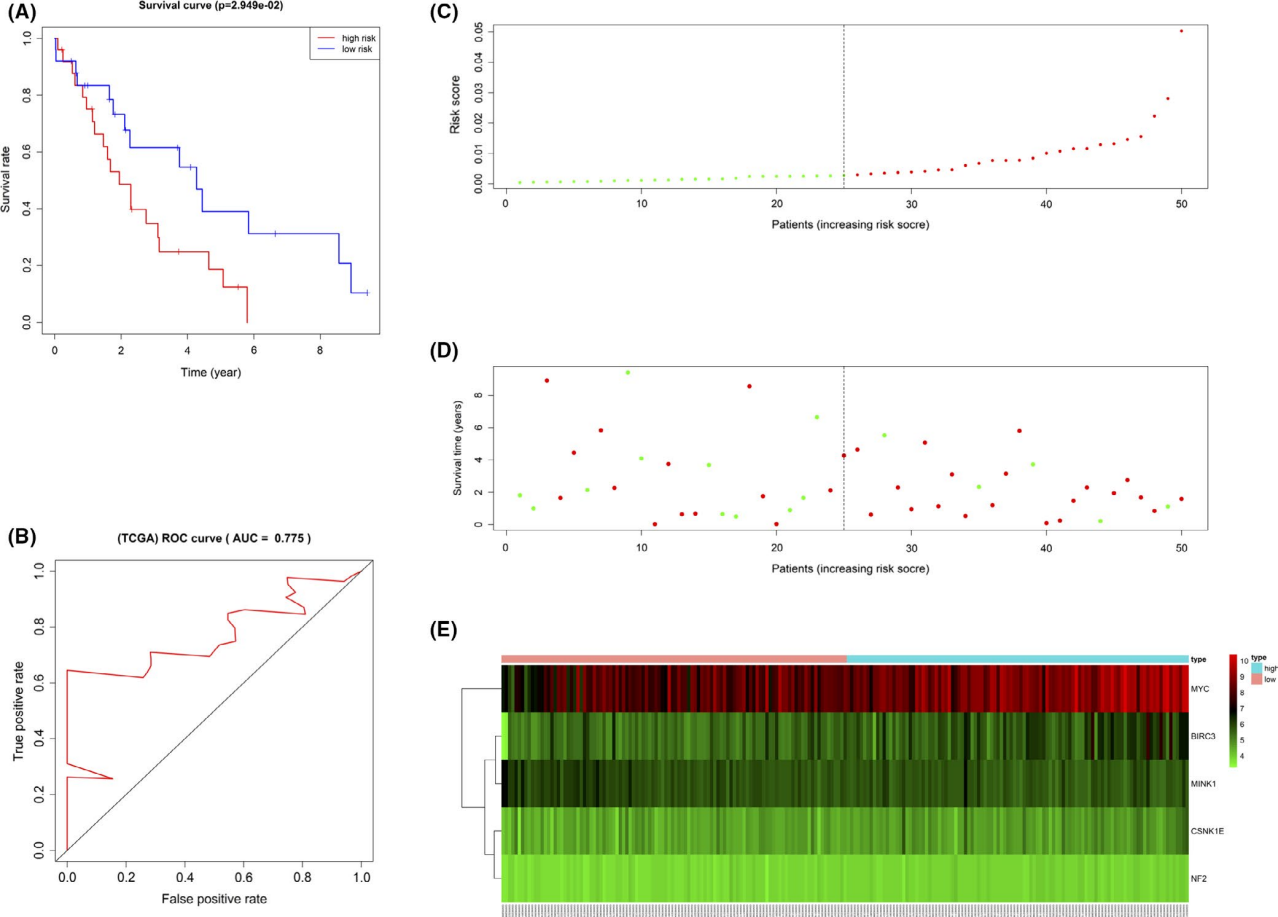
Validation of the HRG‐based prognostic model using PNTAH expression profiles from TCGA‐LIHC. (A) Kaplan–Meier curve analysis of the high‐risk and low‐risk groups. (B) Time‐dependent ROC curve analysis of the prognostic model. (C) The risk score distribution of patients in the prognostic model. (D) Survival status scatter plots for patients in the prognostic model. (E) Expression patterns of risk genes in the prognostic model. PNTAH, paired normal tissues adjacent to HCC; ROC, receiver operating characteristic curve.

The correlation between the risk score and the survival of HCC patients in TCGA‐LIHC was further analyzed to validate the performance of the constructed model. As described above, these patients were classified into low‐ or high‐risk groups based on the median risk score calculated with the prognostic model constructed using the PNTAH expression profiles from TCGA‐LIHC. The median risk score of the TCGA‐LIHC cohort was 0.0028. The survival rates of the two groups were significantly different (*p* = 2.949e‐02) (Figure 6A). In addition, the AUC calculated by the ROC curve was 0.775, indicating that the model predicts HCC patient survival well (Figure 6B). The risk score distribution, the number of patients examined, and the heatmap of the prognostic HRGs in different risk groups are also displayed (Figure 6C–E).

### 3.6 Independence of prognostic model assessment and nomogram development

**FIGURE 7:**
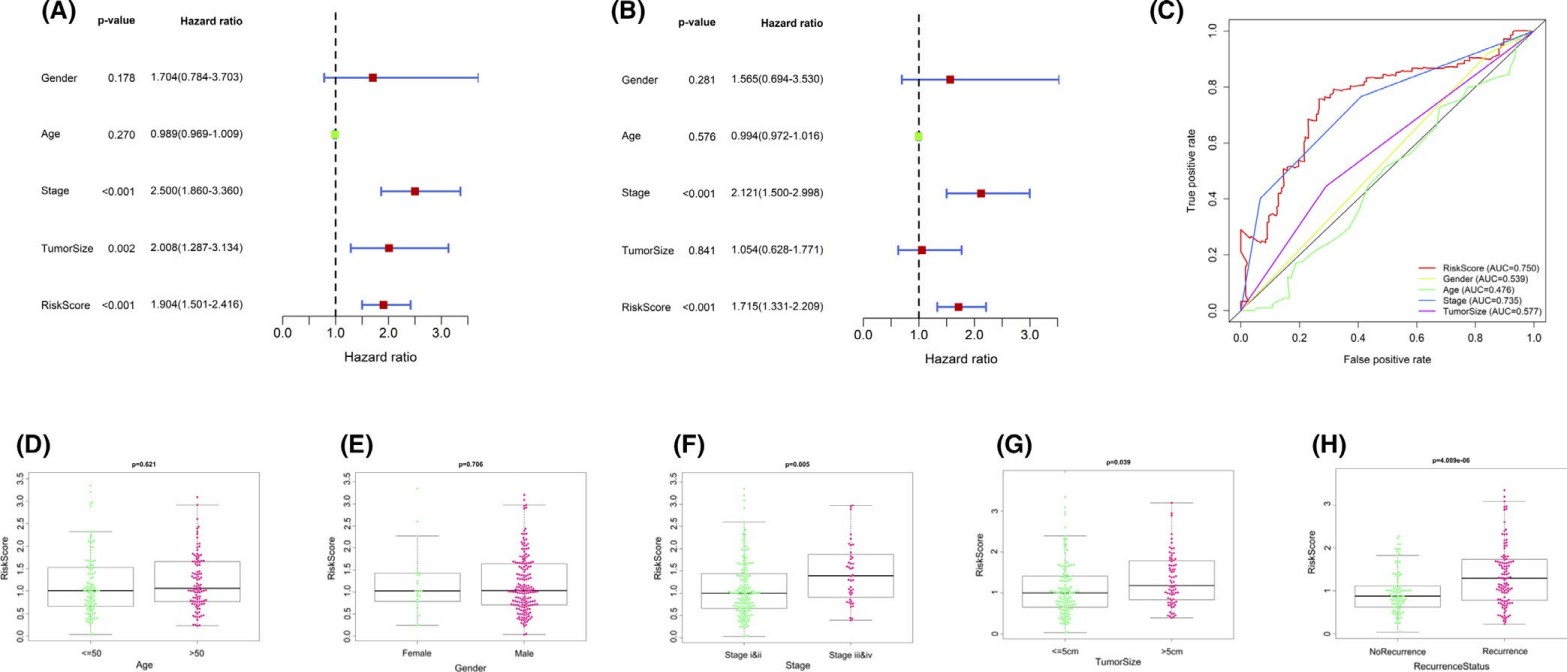
Independence of the prognostic model and correlations with clinical parameters. (A) Univariate Cox regression analysis. (B) Multivariate Cox regression analysis. Age: ≤50 versus >50, gender: male versus female, stage: I/II versus III/IV, risk core: high risk score versus low risk score (median risk score as the cutoff value). (C) Receiver operating characteristic (ROC) curve analysis for the prognostic values of the prognostic model and other conventional clinical parameters. AUC: area under curve. (D) Association with risk score and age. (E) Association with risk score and gender. (F) Association with risk score and tumor stage. (G) Association with risk score and tumor size. (H) Association with risk score and recurrence.

**FIGURE 8:**
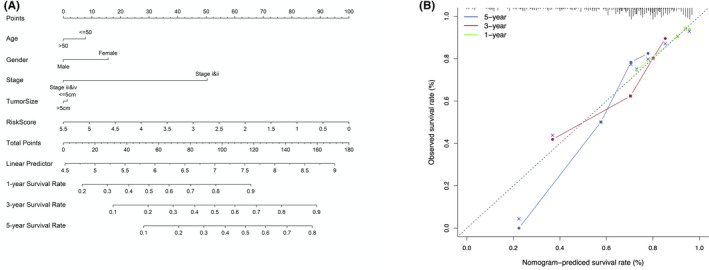
Nomogram with calibration curves for the prediction of prognosis at one, three, and five years. (A) Nomogram for survival rate. (B) Calibration curves.

Multivariate and univariate Cox analyses were performed to explore whether the risk score or other conventional clinical parameters of HCC patients in the GEO cohort were independent risk factors for OS. Univariate Cox analysis showed that advanced stage, larger tumor size, and risk score were risk factors for OS (Figure 7A). However, after multivariate analysis and adjustment for clinical parameters, only the risk score (HR = 1.715, 95% CI = 1.331–2.209, *p* < 0.001) and tumor stage (HR = 2.121, 95% CI = 1.500–2.998, *p* < 0.001) remained as independent prognostic factors for patients (Figure 7B). The ROC curve was plotted to compare the predictive value of the risk score with other clinical parameters. The results indicated that the tumor stage (AUC = 0.735) had the highest predictive value among the conventional clinical parameters (Figure 7C). However, the predictive value of the risk score (AUC = 0.750) was higher than that of the tumor stage. We further analyzed the correlations between the risk score calculated by the model and the clinical parameters in patients with HCC (Figure 7D–H). We found that a high‐risk score was significantly related to larger tumor size (*p* = 0.039), advanced stage (*p* = 0.005), and more recurrence (*p* = 4.009e‐06). To show the relationship between individual predictors and survival rate, a nomogram model was developed based on the data of the GEO cohort and converted to scale within a certain range (Figure 8A). Each parameter (age, gender, tumor stage, tumor size, and risk score) in the map corresponded to a point. The points of all parameters were summed up to obtain a total point, which was used to determine the 1‐, 3‐, and 5‐ year overall survival rates. The C‐index was 0.755, and the calibration curves for the 1, 3, and 5 survival predictions had good linearity (Figure 8B), which meant that the nomogram had favorable calibration.

### 3.7 Verification of the specificity of the five HRGs and the prognostic model using HCC tissue expression profiles

To investigate whether the expression of the five selected HRGs in the HCC tissues also had prognostic value, 232 HCC tissue expression profiles were extracted from the GSE14520 dataset. According to the median expression of each HRG, HCC patients were divided into high‐ and low‐expression groups. Survival analysis showed no statistical difference between the high‐ and low‐expression groups for each HRG (Figure S2A–E). Furthermore, the risk score was calculated by the PNTAH‐derived formula using the expression values of the HRGs in the HCC tissues. HCC patients were divided into high‐ or low‐risk groups based on the median risk score. However, there was no statistical difference in the survival rates of the high‐and low‐risk groups (Figure S2F). Similar results were obtained in analyses of HCC tissue expression profiles from TCGA‐LIHC (Figure S3A–F). The above results indicate that the five selected HRGs and the constructed model are PNTAH‐specific.

## 4 DISCUSSION

HCC is characterized as insidious, showing rapid progress, and a low early diagnosis rate. Due to the heterogeneity of liver cancer, conventional clinical parameters such as age, gender, and stage are often unable to accurately predict clinical outcomes.^15^ In recent decades, many studies have focused on identifying novel biomarkers to promote the prediction of HCC patient survival.^16^, ^17^, ^18^ Based on the advantages reported by these studies, a combination of multiple prognosis‐related genes with conventional clinical parameters for the construction of a prognostic model may demonstrate better performance.^19^, ^20^ However, most studies focused only on HCC expression profiles and ignored PNTAH.

According to the published literature and our findings, the gene expression patterns of NL, PNTAH, and HCC are very different. In fact, as early as 1953, Slaughter et al proposed the “field cancerization” hypothesis, suggesting an intermediate, precancerous state of tumor‐adjacent tissues which are histologically normal but molecularly changed.^21^, ^22^ This theory was supported by later studies. For example, the characteristics of breast cancer genes were found to be differentially expressed in adjacent normal breast epithelium, which might underlie second primaries and local recurrences.^23^ Moreover, epithelial to mesenchymal transition, wound healing, and extracellular matrix remodeling occur in breast cancer‐adjacent normal tissues.^24^ Observations interpreted as reflecting field cancerization have also been made in bladder, colon, and lung cancers.^25^, ^26^, ^27^ Another theory holds that tumor‐adjacent tissues are involved in the formation of a tumor microenvironment that either promotes or suppresses tumor development. A recent study demonstrated that inflammation and immune response‐related gene expression were increased in triple‐negative breast cancer‐adjacent tissues.^28^ Tumor‐adjacent tissues were found to exhibit higher expanded clone ratios of the T cell receptor β‐chain in HCC patients.^29^ Therefore, biomarkers derived from PNTAT may be helpful in predicting prognosis.

Hippo signaling is an evolutionarily conserved pathway that plays an important role in tissue homeostasis.^30^ It has been reported that the control of the liver size relies on the tight regulation of the Hippo signaling activity, and the activation of YAP/TAZ, two main transcriptional activators of Hippo signaling, involved in the repair and regeneration of the damaged liver tissue.^13^ Although many studies have shown that the dysregulation or mutation of Hippo signaling components commonly causes tumorigenesis,^31^, ^32^, ^33^ a recent study demonstrated that experimental overexpression of YAP in peritumoral hepatocytes induced regression of primary liver tumors and metastases.^14^ This finding sheds light on the tumor‐suppressive effect of Hippo signaling in PNTAH. However, a prognostic model based on HRGs and PNTAH has not been previously established.

In this study, we used the expression profiles of PNTAH from GEO to construct an HRG prognostic model and validated this model using PNTAH data from TCGA‐LIHC. First, we identified 76 genes from four Hippo‐related canonical pathways as HRGs. Univariate Cox regression analysis identified 14 genes closely related to the survival of patients with HCC. LASSO and multivariate Cox regression analyses were used to select five key HRGs (BIRC3, CSNK1E, MINK1, MYC, and NF2) to construct the prognostic model. High expression levels of BIRC3, CSNK1E, MYC, and NF2 in PNTAH were associated with poor prognosis in HCC patients, while MINK1 expression was associated with a good prognosis. We divided patients into high‐ or low‐risk groups based on the median risk score calculated by the model formula and found that the high‐risk group had a lower survival rate. The AUC calculated by the ROC curve was 0.750, indicating that the model could predict the survival of HCC patients. We then confirmed that the risk score was an independent prognostic indicator after adjusting for other clinical parameters. ROC curve analysis demonstrated that the risk score had a better predictive value than other clinical parameters. Finally, we established a nomogram that predicted the survival of HCC patients well. The use of this tool could help clinicians dynamically assess a patient's prognosis based on different levels of clinical parameters and implement more targeted interventions accordingly. Furthermore, we also observed a similar trend in the survival analysis and ROC curve analysis of an independent dataset from TCGA, which further confirmed the reliability of this prognostic model. Given that PNTAH might be a precancer state, the five HRGs and the constructed model could have better prognostic performance in HCC tissues. We investigated the prognostic ability of the five HRGs and constructed a model using the expression profiles of HCC tissues from GEO and TCGA‐LIHC. Interestingly, the results showed that neither the five HRGs alone nor the constructed model had prognostic value in HCC tissues. Thus, we concluded that the prognostic model based on HRGs had a prognostic value specific for PNTAH.

We further explored five selected HRGs. NF2 is a well‐established tumor suppressor and an essential upstream regulator of Hippo signaling.^34^, ^35^, ^36^ However, our data showed that NF2 was highly expressed in HCC tissues, and the high expression level of NF2 in PNTAH was associated with poor prognosis in HCC patients. This paradox may be explained by the mutations in NF2. NF2 mutations have been observed in many tumors, which encode Merlin proteins that cannot inhibit tumorigenesis.^37^, ^38^, ^39^ The loss‐of‐function of NF2 severely compromises Hippo signaling activity through the major effector YAP, resulting in hepatomegaly and liver cancers.^40^ To date, there have been no reports on the use of NF2 as a prognostic factor in cancer. MYC is a downstream gene of Hippo signaling and a well‐known oncogene.^41^, ^42^, ^43^ Previous studies have noted MYC expression as a poor prognostic marker in various cancers, such as breast cancer, osteosarcoma, and pancreatic ductal adenocarcinoma.^44^, ^45^, ^46^ Our results showed that high expression of MYC in PNTAH predicted poor prognosis of HCC patients, which could be explained to a certain extent by the “field cancerization” theory. BIRC3 belongs to the inhibitor of apoptosis proteins family, which has been shown to be regulated by Hippo signaling.^35^, ^47^ It has been reported that BIRC3 is associated with a poor prognosis in gliomas.^48^ Both CSNK1E and MINK1 are serine/threonine protein kinases belonging to the casein kinase I protein family and the germinal center kinase family, respectively.^49^, ^50^ A previous study identified CSNK1E as a new temporal regulator of the Hippo pathway.^51^ MINK1 is a known upstream factor that regulates Hippo signaling through the LATS1/2‐YAP/TAZ interaction.^52^ The roles of CSNK1E and MINK1 in a variety of cancers have not yet been investigated.

Some limitations of this study should also be considered. First, while the expression profiles were downloaded from the GEO and TCGA databases, the sample size was not large enough. Second, we only focused on the mRNA levels of these genes, and the five genes at the protein level should be further investigated. Third, the results of our study are descriptive, and the potential molecular mechanisms of these five genes warrant additional functional experiments. In addition, it has not been determined whether the median risk score we chose in the GEO and TCGA databases can be used as the threshold in real‐world clinical practice to identify high‐risk and low‐risk patients.

## 5 CONCLUSIONS

In the present study, we constructed and validated a prognostic model using PNTAH expression profiles, and this model could predict the survival of patients with HCC. The differentially expressed HRGs may provide a new perspective for the elucidation of the molecular mechanisms of HCC.

## CONFLICTS OF INTEREST

The authors have no conflicts of interest to declare.

## AUTHOR CONTRIBUTIONS

(I) Conception and design: Y Zeng; (II) Administrative support: H Ren; (III) Provision of study materials or patients: QB Pan; (IV) Collection and assembly of data: BN He, N Yang, YT Zhang, HY Yuan; (V) Data analysis and interpretation: QB Pan, FB Qin; (VI) Manuscript writing: All authors; (VII) Final approval of manuscript: All authors.

## ETHICAL STATEMENT

The authors are accountable for all aspects of the work in ensuring that questions related to the accuracy or integrity of any part of the work are appropriately investigated and resolved. All data were publicly available and downloaded from online databases; therefore, this study did not require additional ethical approval.

## Supporting information

Fig S1

Fig S2

Fig S3

## Data Availability

The data that support the findings of this study are available from the corresponding author upon reasonable request.
