# Supplementary figures and images for: Normal tissue adjacent to tumor expression profile analysis developed and validated a prognostic model based on Hippo‐related genes in hepatocellular carcinoma
Source: Cancer Med. 2021 Apr 4;10(9):3139–52. doi: 10.1002/cam4.3890 (PMC8085948; doi:10.1002/cam4.3890)

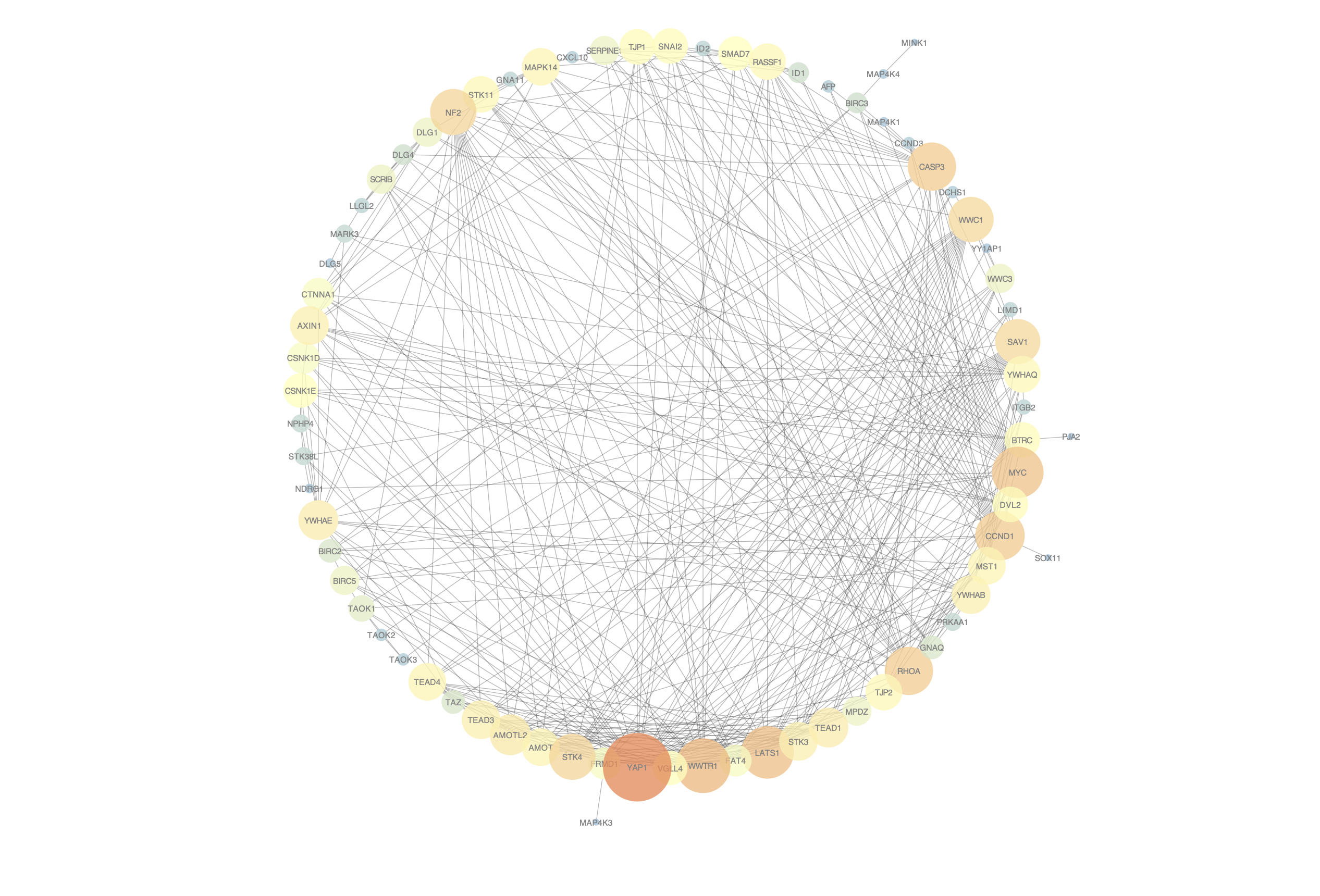

Supplement: Supplementary file 1 — Fig S1 [file CAM4-10-3139-s002.tiff]

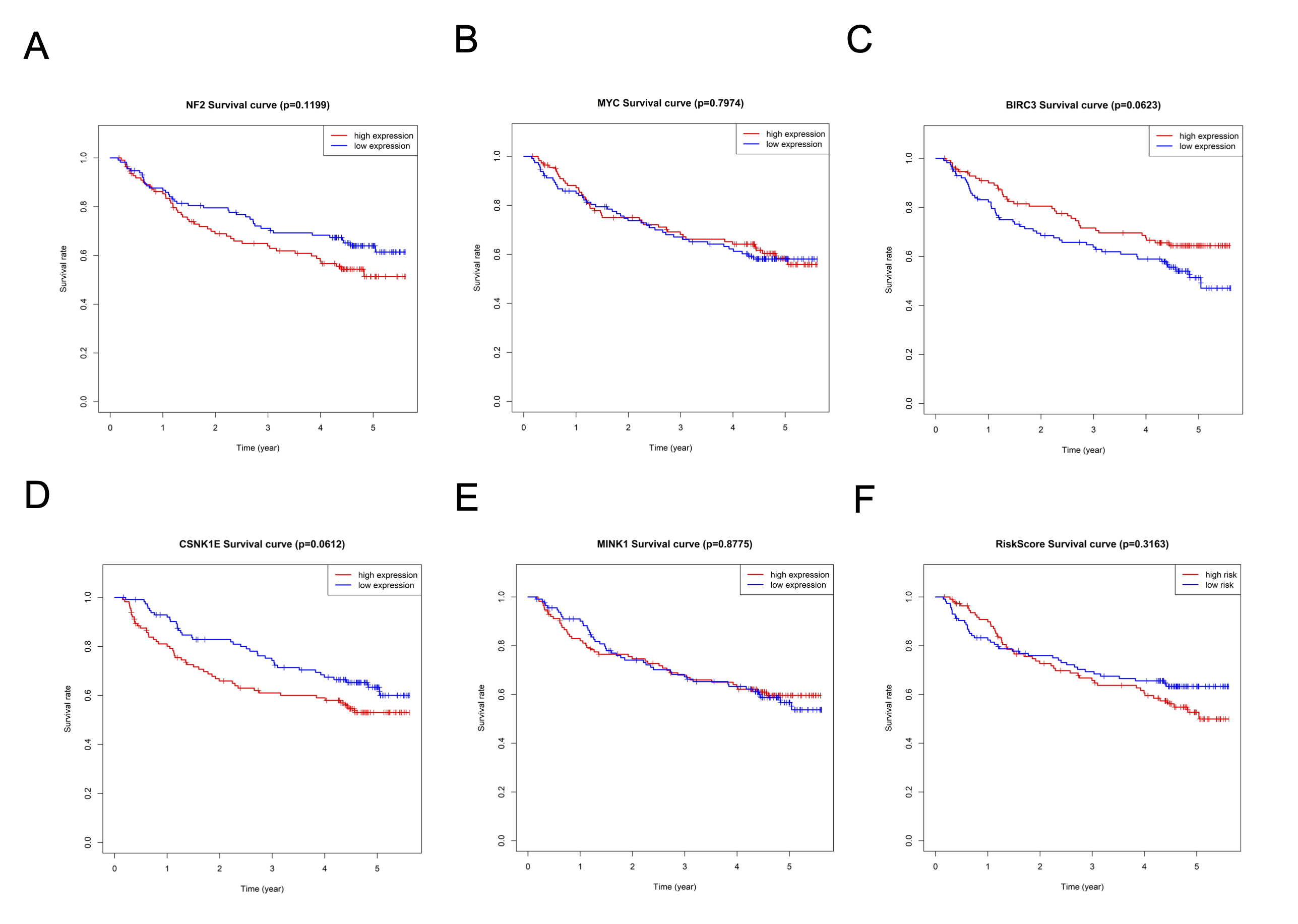

Supplement: Supplementary file 2 — Fig S2 [file CAM4-10-3139-s001.tiff]

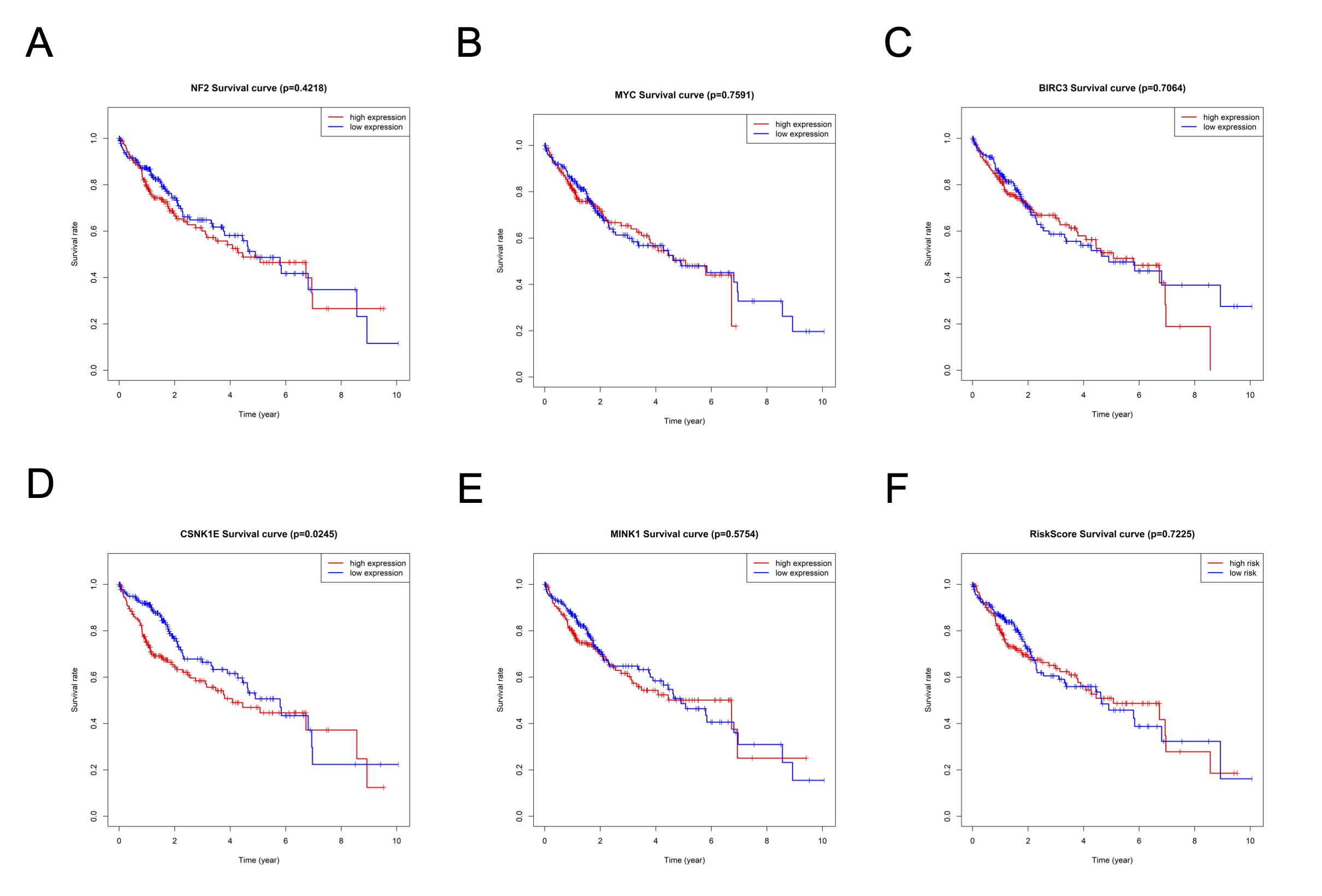

Supplement: Supplementary file 3 — Fig S3 [file CAM4-10-3139-s003.tiff]
